# Co-prioritization of mental health recovery outcomes and scales for community mental health centers in Peru

**DOI:** 10.1186/s12913-025-13140-7

**Published:** 2025-09-01

**Authors:** Victoria Cavero, Francisco Diez-Canseco, Antonio Bernabé-Ortiz, Lisette Gamboa Galvez, Noelia Cusihuaman-Lope, Marcia Sanchez-Monge, Maria Lazo-Porras

**Affiliations:** CRONICAS Center of Excellence in Chronic Diseases, Universidad Peruana Cayetano Heredia, Lima, Peru

**Keywords:** Mental health, Health services, Community mental health centers, Outcome assessment, Participatory research, Co-prioritization

## Abstract

**Background:**

Mental health recovery outcomes are scarcely used and monitored in low- and middle-income countries, despite their importance on assessing the results of the care provided and potential areas of improvements. In Peru, the Mental Health Directorate (MHD) monitors mental health services mainly based on the number of people served and not on the improvements or recovery of their patients. This study aims to conduct a co-prioritization process with key stakeholders to introduce recovery outcomes and scales in community mental health centers (CMHC) in Peru.

**Methods:**

The co-prioritization methodology combined periodic meetings with MHD’s heads; a literature search and conversations with nine international mental health experts; and eight participatory workshops with Peruvian key stakeholders (policymakers, CMHC workers, and patients). All the information was analyzed using matrices and thematic analysis.

**Results:**

Nine outcomes were identified in the literature search and conversations with mental health experts, and five outcomes were finally prioritized by key stakeholders. After revision and discussion of several scales for each outcome, two scales were prioritized by all stakeholders: WHODAS-12 and DIALOG. Policymakers, workers, and patients prioritized three of these outcomes: psychosocial functioning, quality of life, and psychiatric symptoms. The first two were the most important for the three groups, whereas symptoms were more important for policymakers and workers than for patients. Additionally, patients prioritized emotional balance and personal growth, two emerging outcomes that were not identified in our previous literature search and conversations with experts. Scales were prioritized based on their relevance, usability, and feasibility to integrate them into the CMHC routines: WHODAS-12 to assess psychosocial functioning and DIALOG for quality of life. Stakeholders did not agree on a single scale to assess symptoms due to the large array of symptoms that their patients present, and no scale was assessed for emotional balance and personal growth since they only emerged in the final set of workshops.

**Conclusion:**

Based on a participatory methodology, key stakeholders at different levels of the Peruvian mental health system prioritized five recovery outcomes to use routinely in CMHC: psychosocial functioning, quality of life, psychiatric symptoms, emotional balance, and personal growth. The first two were deemed as the most important for all stakeholders; and the latter two were novel outcomes that emerged from patients. Two scales were selected to assess the first two of these outcomes. Defining a scale for the latter three outcomes and test their use in CMHC routines remain as pending tasks.

**Supplementary Information:**

The online version contains supplementary material available at 10.1186/s12913-025-13140-7.

## Introduction

Many low- and middle-income countries (LMICs) have made mental health reforms over the last few decades, but these reforms do not necessarily lead to better outcomes for their patients [1], such as better quality of life, improved psychosocial functioning, or reduction of symptoms [2]. Although many high-income countries have analyzed the introduction of outcome measures and scales in their routines [2–5] we could not identify studies exploring this in LMICs. Indeed, a great gap in the global mental health field is the lack of monitoring and accountability of public mental health services, highlighting the importance of defining indicators that are routinely collected and comparable to other contexts [6].

Here, we present the experience of Peru, a middle-income country which has implemented a community mental healthcare system [7–10], and adopted recovery-oriented interventions [11]. In Peru, community mental health centers (CMHC) are the main facilities responsible for providing specialized outpatient care to people with severe mental disorders or psychosocial problems [12]. The implementation of these facilities began in 2013 [13], and nowadays, there are nearly 300 CMHC throughout the country, composed of multidisciplinary teams who provide a set of mental health care packages for people with different mental and psychosocial conditions [14].

The fulfillment of these health care packages is monitored by the results-based budgeting program (PPR, in Spanish) for mental health of the Ministry of Health (MoH), which aims to establish and supervise that the CMHC comply with a proportion of people receiving these packages [15, 16]. This is reflected, for example, in the proportion of people who have been screened and received care in a timely manner [16]. However, similar to other countries [1], this monitoring still lacks specific outcomes, routine measurement, and reports of such outcomes to evaluate improvements in their patients’ mental health. The current implementation of the mental health module of the electronic health records in CMHC [10] can change this. Since 2018, the primary care system in Peru is moving from paper-based health records to electronic ones [17] enabling the standardization of the information gathered in different centers. This represents a good opportunity to introduce the measurement of recovery outcomes as part of the CMHC routines nationwide but considering adequate technological conditions, acceptance of key stakeholders and assure optimal training and support [18]. To increase the likelihood of a successful implementation of mental health interventions, the participation of different stakeholders involved in the provision of these services, such as patients and health workers, is essential [19]. Thereby, we conducted a participatory process with the Mental Health Directorate (MHD) at the MoH, to collect various points of view from policymakers, CMHC workers and patients to co-prioritize the best recovery outcomes and scales to measure them that could be introduced routinely in CMHC. This study’s results will help the MHD to incorporate an initial common set of recovery outcomes and scales into the regular care of CMHC along the country.

## Methods

### Design

This study, conducted throughout 2023, involved the participation of CMHC patients and workers, as well as national mental health policymakers, who, based on their different experiences, could inform and provide recommendations to select and adjust the recovery outcomes and scales to the Peruvian context. Our design followed the principles of the experience-based co-design, a collaborative approach that gather together patients and workers to improve the provision of mental health care [20]. Yet, we added some additional changes since this method has been widely used in hospital-based settings and rarely in community-based ones [21].

Thereby, we used a mixed-method approach, through multiple components [22]. First, continuous *meetings with heads from the Mental Health Directorate (MHD)* throughout the co-prioritization process. Second, a *literature search* and *conversations with international mental health experts* to collect information about mental health recovery outcomes and scales used worldwide. Finally, *co-prioritization workshops with local key stakeholders* (policymakers from the MHD, CMHC workers, and patients) to select a set of mental health recovery outcomes and scales to be used in the CMHC of Peru. Additional file 1 shows the timeline of the different activities.

### Co-prioritization components

#### Component 1: meetings with heads from the MHD 

The research team held regular meetings with heads from the MHD who led the initiative to introduce mental health recovery outcomes in the CMHC. The meetings lasted around 90 min, were conducted in the MoH facilities or online, and were held among the research team and the MHD heads, to define the methods, reflect on the main results of each component, and help in their synthesis. The research team took notes during these meetings, which were then summarized and used to guide the next meetings as well as incorporate the decisions and suggestions made in the next components.

#### Component 2: Iiterature search and conversations with mental health experts

We conducted a literature search to identify mental health recovery outcomes and scales used worldwide, and identified a systematic review [2] that provided a global scope of potential mental health outcomes and scales that could be used in the CMHC of Peru. Then, we reached mental health clinicians and/or researchers who worked in or with LMICs, to gather their experiences using mental health recovery outcomes and scales in their countries. We conducted online meetings with each expert using an interview guideline detailed in Additional file 2 with common topics but tailored to each participant’s experience. Main topics were: which outcomes and scales they recommended to assess recovery in CMHC patients, what are the gold standards to assess recovery in mental health patients, what outcomes and scales are used in their countries and/or other LMICs, and recommendations on how to use them in public health services. The notes taken during each meeting were then gathered together and summarized in a short report with three main topics: (1) Suggested outcomes; (2) Suggested scales; and (3) Recommendations to assure the use of these scales in public mental health services.

#### Component 3: workshops with key stakeholders 

We conducted eight workshops with three groups of stakeholders: two workshops with policymakers, two with CMHC workers, and four with CMHC patients. Even though most workshops were conducted in Lima, the capital city of Peru, the research team sought to gather the geographical and cultural diversity of the country by inviting workers and patients from different regions, representing cities from the north, center, and south of the country, as well as the coastal, highlands, and Amazon regions.

Workshops were designed based on the project aims and with the constant feedback of senior researchers from the research team (ML has experience in participatory methods, and FDC in qualitative mental health research), and the MHD heads. Tailoring activities to each group, workshops sought to collect the experiences of participants with recovery in CMHC patients, the mental health outcomes, scales that could be used to assess this recovery, and the conditions to assure their proper use in CMHC in Peru.

##### Workshops with policymakers

First, the research team conducted two two-hour workshops with policymakers, who were members of the MHD in charge of the country’s mental health policies. The first workshop consisted of a presentation about recovery outcomes identified in the literature and recommendations received by international experts. Policymakers prioritized individually a set of recovery outcomes to use in CMHC; and then, in groups, they explained their reasons to select them. In the second workshop, they selected up to three scales per outcome based on their relevance, usability, and feasibility to administer them in CMHC.

##### Workshops with CMHC workers 

Second, a two-day six-hour workshop was held with CMHC psychologists, nurses, doctors, and social workers from various regions. On the first day, participants discussed in groups how they identify recovery in their patients and were then informed about the outcomes prioritized by policymakers. Workers’ responses aligned to three recovery outcomes identified in the literature, which agreed with three of the prioritized by policymakers (psychosocial functioning, symptoms, and quality of life). The second day focused on evaluating and co-selecting scales for measuring these outcomes. Participants reviewed instruments individually and in groups, considering their relevance, usability, and feasibility to integrate them into the CMHC routines. The workshop concluded with a virtual voting to select the three best scales for each outcome.

##### Workshops with CMHC patients

Third, six-hour workshops with CMHC patients were conducted in Lima (one pilot and one workshop, coastal and capital city), Arequipa (one workshop, highlands city), and Ucayali (one workshop, Amazon city), involving individuals with common mental disorders, severe mental disorders, substance abuse disorders, and psychosocial problems. The workshops aimed to gather patients’ experiences of improvement and recovery, identify and prioritize recovery outcomes, and review selected scales with patients. Activities included group discussions on patients’ recovery journeys, creating graphs to illustrate their progress and challenges, and listing desired future improvements. Each patient then prioritized five key recovery outcomes and explained their importance in a plenary session. Finally, they reviewed the scales prioritized by CMHC workers, providing feedback on their clarity and ease of understanding.

To assure fidelity, each workshop was designed using a matrix and detailed text to guide the facilitators on how to conduct them. A summary of these matrices can be found in Additional file 3. Each matrix presented the objectives, activities, materials, and roles; and the text described how to implement each activity.

Workshops were designed in collaboration with the heads of the MHD, who provided their feedback on the methodology and supported the recruitment of participants. The MHD heads sent formal invitations to the MHD policymakers and to the local health directions of 10 Peruvian regions to allow the research team to reach and recruit potential participants.

### Participants

Different participants were involved in each component. Five MHD heads participated in the first component, who were involved in the CMHC monitoring. Then, nine international experts were involved in the second component. The research team had previous experience working with four of these experts (two from the UK and two from Colombia), and they recommended reaching the following experts (one from Colombia, three from Chile, and one from Zimbabwe). These experts were researchers and/or clinicians with extensive experience in mental health systems’ functioning in Latin America or other LMICs. Finally, in the third component, 12 policymakers from the MHD participated, 22 CMHC workers from ten different regions and professions, and 30 patients from three different regions and diagnoses. Table 1 shows the number, inclusion/exclusion criteria, and demographics of the participants of each component.

**Table 1:** Participants’ characteristics per component

Components	Inclusion/exclusion criteria	Demographics
Component 1: Meetings with heads from the MHD	- Heads from the MHD involved in the monitoring of CMHC provision of care	- Total: 05 o Male: 01 o Female: 04
Component 2: Conversations with international mental health experts	- Researchers and/or clinicians with extensive experience in mental health - Well-informed about the mental health systems’ functioning of Latin America and low- and middle-income countries	- Total: 09 o Male: 05 o Female: 04 - Country: Colombia (3), Chile (3), United Kingdom (2) and Zimbabwe (1)
Component 3: Workshops with policymakers	- Have been working in the MHD for at least 06 months - Well-informed about the functioning of the CMHC	- Total: 12 o Male: 04 o Female: 08
Component 3: Workshops with CMHC workers	- CMHC clinicians (e.g. psychiatrists, family doctors, psychologists, nurses, and social workers) - At least 02 years working in a CMHC - Being proactive and open to share their ideas - Working in a CMHC from one of the 10 selected regions for the study	- Total: 22 o Male: 04 o Female: 18 - Professions: Psychiatrists, family doctors, psychologists, nurses, and social workers - Regions: Amazonas, Ancash, Arequipa, Ayacucho, Junín, La Libertad, Lima, Piura, San Martín, and Ucayali
Component 3: Workshops with CMHC patients	- Adults older than 18 - Have been receiving care in a CMHC for at least six months - Have a diagnosis of mental disorder or psychosocial problem - Being in capacity of participate in a group workshop and provide informed consent	- Total: 30 o Male: 09 o Female: 21 - Diagnoses: common mental disorders (e.g. depression, anxiety); severe mental disorders (e.g. schizophrenia, bipolar disorder); substance abuse disorders (e.g. alcohol abuse); and psychosocial problems (e.g. victims of violence) - Regions: Arequipa, Lima, and Ucayali

*CMHC* Community mental health centers, *MHD* Mental Health Direction of the Ministry of Health

### Analysis

All sessions were audio recorded and transcribed verbatim, whereas physical materials, such as cards and papers, were stored in secure places and all data retrieved to Excel sheets. Through figures, tables, and quotes, the research team organized and synthetized all the workshops data, using thematic analysis [23]. The research team held continuous meetings to discuss the findings.

### Ethics

This study was conducted in accordance with the Declaration of Helsinki and was approved by the IRBs of Universidad Peruana Cayetano Heredia (Letter CIEI-396-34-22) and of King’s College London (Letter HR/DP-22/23-33776). All participants signed a written informed consent form before participating in any activity.

## Results

**Fig. 1:**
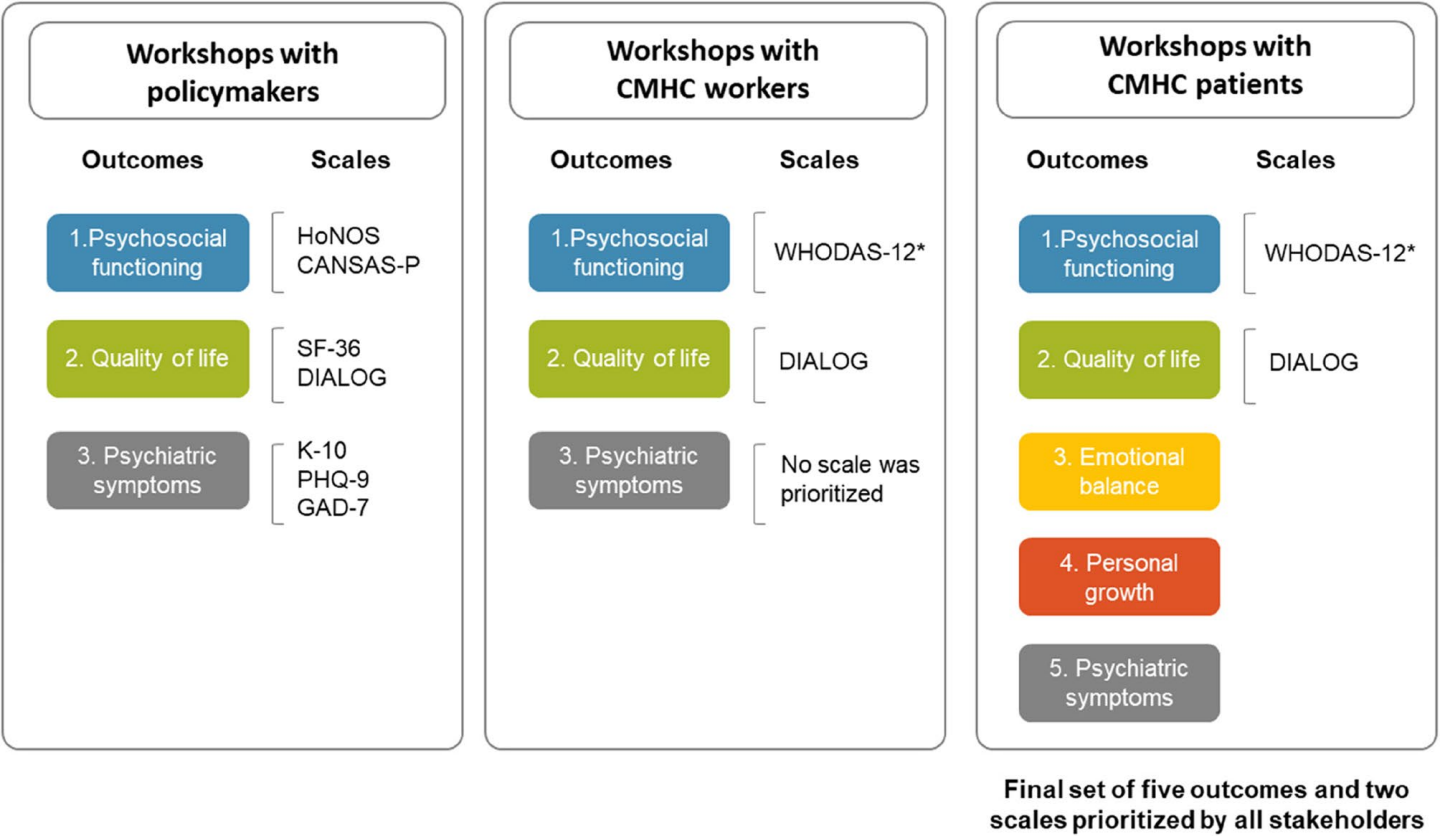
Flow of outcomes and scales prioritized by key stakeholders. *WHODAS-12 was added by MHDs’ heads of the Mental Health Direction of CMHC community health centers

Table 1 shows the characteristics of participants in each component. Here, we present the results of each component, integrating the voices of all participants involved. A final set of five outcomes and two scales were finally prioritized by all stakeholders (See Fig. 1):

### Component 1: meetings with heads from the MHD

Five meetings were conducted with MHD’s leaders. In the first meeting, the structure of the co-prioritization activities and profiles of stakeholders involved were decided. In the following meetings, we discussed the objectives, methods, and findings of Components 2 and 3, and agreed the next steps. Sometimes, the MHD heads suggested changes. For example, in the third meeting, they suggested including WHODAS-12 as an extra scale to assess psychosocial functioning and moving HoNOS for symptoms assessment. These changes were made based on the scales’ content and perceived utility for the CMHC.

### Component 2: literature search and conversations with mental health experts

In the literature searching, we found a systematic review [2] that covered our research aims of identifying recovery outcomes and scales used worldwide. Although these authors mentioned in a previous manuscript [32] that they sought articles published in English, in peer-reviewed journals, between 2000 and 2018, and from around the world; they only included studies from North America, Europe and Australia. Their results showed nine mental health outcomes and divided scales in two groups: patient-reported outcome measurement (PROMs), which assess patients’ feedback; and routine outcome measures (ROMs), which assess perspectives from health workers and caregivers. The review listed over 100 scales distributed across the nine outcomes. We pre-selected 19 scales based on the most commonly used according to the review, their availability in Spanish, their extension (prioritizing the shorter ones), and their validations in settings similar to Peru. Table 2 shows the nine outcomes pre-selected by the research team, their definitions, and the 19 scales.

**Table 2:** Pre-selection of mental health outcomes and scales, divided by responders (patients and health workers)

Mental health recovery outcomes	Definitions	Scales	
		Responded by patients (Patient-reported outcome measurement/Routine outcome measures)	Responded by health workers (Routine outcome measures)
1. Psychosocial functioning	A person’s ability to manage daily responsibilities and interact with others and society effectively. It can be domain-specific (e.g., marital, social, occupational, academic, or physical functioning) and may sometimes be evaluated objectively [24].	1. CORE-OM 2. OQ-45	3. HoNOS
2. Quality of life	An individual’s perception of their life, shaped by cultural values and influenced by their goals, expectations, and concerns. It includes personal health, relationships, education, work, social status, wealth, security, freedom, self-determination, social connection, and surroundings [25].	4. SF-36 5. EQ-5D	6. MANSA
3. Needs assessment	The identification of unmet healthcare needs and making necessary adjustments to address them [26].	7. CANSAS-P	
4. Symptoms and symptoms severity	Clinical manifestations may be objective (physician-observed) or subjective (patient-reported) [27].	8. K-10 9. PHQ-9 10. GAD-7	11. CGI
5. Satisfaction with services	Client satisfaction encompasses quality care aspects defined by health professionals (e.g., clinical practices, drug availability) and by the community (e.g., waiting period, provider interaction) [28].	12. CTM-15 13. Client Satisfaction Questionnaires 14. VSSS	
6. Disability and functional impairment	A condition (impairment) that restricts activity and interaction with the environment. Functional impairments include lost or limited body function, like persistent pain or stiff joints [29].		15. GAF 16. LSP-16
7. Risk assessment to alcohol abuse	Questionnaires that evaluate unhealthy alcohol consumption and the risk of alcohol use disorder (AUD), helping to classify its severity (mild, moderate, or severe) [30].		17. FACE Risk Profile 18. AUDIT: Interview Version
8. Shared decision-making	A process where clinicians and patients collaborate to select tests, treatments, or management plans based on evidence and patients preferences, supported by information on options and outcomes [31].		19. SDM-Q
9. Clinicians’ attitudes and training	Skills of providers, improvements in their service quality, their work impact, the therapeutic alliance they develop with patients and their education, monitoring and clinical feedback needs [2].		20. WAI

*CORE-OM* The Clinical Outcomes in Routine Evaluation-Outcome Measure, *OQ-45* Outcome Questionnaire-45, *HoNOS* Health of the Nation Outcome Scales, *SF-36* Short Form-36 Health Survey, *EQ-5D* European Quality of Life 5 Dimensions, *MANSA* Manchester Short Assessment of Quality of Life, *CANSAS-P* The Camberwell Assessment of Need Short Appraisal Schedule, *K-10* Kessler Psychological Distress Scale, *PHQ-9* Patient Health Questionnaire-9, *GAD-7* Generalised Anxiety Disorder Assessment, *CGI* Clinical Global Impression, *CTM-15* Case Transitions Measure, Client Satisfaction Questionnaires, *VSSS* Verona Service Satisfaction Scale, *GAF* Global assessment of function, *LSP-16* Life Skills Profile, FACE Risk Profile Functional Analysis of Care Environments, *AUDIT* Interview Version, Alcohol Use Disorders Identification Test, *SDM-Q* Shared Decision Making Questionnaire, *WAI* Working Alliance Inventory

Likewise, we conducted individual virtual meetings with nine international mental health experts. According to our interviewed experts, no country in Latin America uses mental health recovery outcomes to assess their public health services. They were very satisfied that this initiative came from the Peruvian government itself.

Experts recommended a series of outcomes that could reflect the recovery of mental health patients. Additional file 4 shows all these outcomes, grouped according to the outcomes found in the systematic review of Component 2. The outcomes most frequently mentioned by the experts were quality of life, psychiatric symptoms, working or studying, interpersonal relationships, living independently, and treatment adherence. For measurement scales, they recommended the Clinical Global Impression (CGI) for its simplicity, relevance to patients’ experiences, and non-reliance on diagnosis, as well as the DIALOG scale, tailored to community-based services. Conversely, they were not in favor of using the Health of the Nation Outcome Scale (HoNOS) for its complexity and unclear interpretation. Experts stressed the importance of scales being mandatory, quick, and easy to apply, suggesting a gradual integration to avoid overwhelming service workers. They also suggested starting with a few scales and then gradually incorporating more, to prevent workers from using them or just filling in mechanically.

To ensure a successful implementation of the new scales, experts advised using outcomes already familiar to CMHC and providing incentives, such as monetary rewards or comparison with other clinics without blaming. They emphasized the need for monitoring and granting workers access to results to prevent scales from becoming burdensome. Additional recommendations included leveraging electronic health records for efficiency and recognizing that chronic mental health patients in community settings may plateau in recovery rather than show continuous improvement. Experts also highlighted issues like licensing fees for scales and the risk of falsified data if unrealistic improvement expectations persist. These insights were shared with policymakers and CMHC staff to guide practical and sustainable implementation.

### Component 3: workshops with key stakeholders

In this section, we report the results of the eight workshops conducted with policymakers, CMHC workers and patients.

#### Participants’ experiences and expectations with recovery

The three groups of participants shared that recovery in mental health patients was a process rather than a goal, and it was dynamic; meaning that they could improve, then relapse, and then recover again. Recovery process depends on the diagnosis and its severity. For instance, for people with psychosis or schizophrenia, the expected outcome was to control their symptoms rather than to eliminate them; whereas for those with depression or anxiety, symptom reduction or elimination was expected.

I not only aspire to recovery, but I feel that I have it right now, and I have to sustain it. (Patient with substance abuse, Southern Lima)

Some policymakers and workers mentioned that the “Continuity of care program”, provided by CMCH to a subgroup of severe cases, assess recovery in their patients. These participants suggested having outcomes and scales to assess recovery for all CMHC patients. They also reflected that finishing a care package would not mean having reached recovery, since many patients could need more packages to feel recovered. Indeed, workers mentioned that they open new care packages if the patient still needs care for the previous or a new condition.

Sometimes a (care) package can be completed, but it does not necessarily mean that the patient has recovered, and we are interested in the patient recovering. Having the ability to live well, being more independent; even if they have some symptoms, it does not prevent them from developing a healthy life. (Female policymaker)

Importantly, some patients said that they would feel recovered once the CMHC discharged them; whereas others expected to be discharged but still continue receiving care (pharmacological or psychological) to maintain their recovery and prevent relapse.

Participants also shared that recovery was more than solely the reduction of psychiatric symptoms, but with some differences in their perceptions. Workers, especially physicians, gave more relevance to the reduction of symptoms than the other groups, mentioning that it was the most immediate outcome to achieve, that they could directly influence their achievement (in comparison to psychosocial functioning and quality of life, which were seen as broader and in the long-term), and even though not sufficient, it was necessary. Yet, policymakers and patients mentioned that even if symptoms did not improve, recovery could be reached when patients return to their previous activities, enjoy their lives, and integrate into society by working, studying, and having healthy relationships. Indeed, patients with severe mental disorders and addictions mentioned that they did not seek the reduction of symptoms but their control, assuming that symptoms may not disappear, but they can be handled.

Policymakers and workers agreed that health workers are usually trained to focus on the reduction of symptoms, which is why the MHD heads emphasized the importance of workers paying attention to other aspects of patients’ lives. Importantly, patients highlighted the importance of recognizing their diagnosis, having personal goals, and wanting to receive treatment as part of their recovery process, since they provided them hope, purpose and skills to maintain the emotional state achieved and prevent relapses and discomfort.

Most patients aspired to recovery and deemed it as a goal to achieve and maintain. They identified various motives for recovery, such as the support of their family and friends, the care provided by professionals and/or the treatment offered by CMHC, their religions, and their own self-determination to feel better.

In my case, I put the highest percentage because it depends on me (…). The confidence and commitment that you must have for your recovery depends on the support of your family, or of your specialist, but more important is your own because it depends on you to move forward.” (Patient with schizophrenia, Northern Lima)

We also identified some differences among patients about what they valued the most in their recovery. Those with severe mental disorders, such as schizophrenia, prioritized their psychosocial functioning and autonomy; those with addictions prioritized being able to work or study; whereas those with depression, anxiety, and victims of violence prioritized having an increased self-esteem, trusting themselves, being resilient and optimistic.

However, two patients stated that they did not see their recovery as possible. One of them, because she had not experienced improvement and perceived these efforts as repetitive and exhausting; and the other, an older woman living alone, because she lost hope and said that she continues taking her medications out of obligation.

To be honest, I don’t think I can improve my mental health anymore. I’m tired of taking so many pills, I’m tired of psychiatrists always telling me the same thing, I’m tired of them finding something else for me every time. So I just don’t want to, I’m not even interested in fitting in, I’m not even interested in fitting into society anymore. (Patient with bipolar disorder, Southern Lima)

#### Co-prioritized mental health recovery outcomes 

The three groups of stakeholders shared with us what outcomes could help them assess the mental health recovery of CMHC patients. Three outcomes were prioritized by policymakers, workers and patients: Psychosocial functioning, quality of life, and symptoms. Additionally, policymakers also prioritized shared decision-making, as an evidence of the autonomy that a recovered patient can gain, but this was excluded by MHD heads for considering it more as a quality outcome of health services rather than a recovery outcome. They also prioritized needs assessment, but they merged it with psychosocial functioning because the needs assessed were similar to the dimensions of psychosocial functioning. Likewise, patients added “emotional balance” and “personal growth”, based on their own experiences and expectations.

All these results were analyzed in meetings with the MHD heads, and five outcomes were finally prioritized: Psychosocial functioning, quality of life, symptoms, emotional balance, and personal growth. Table 3 shows the way in which stakeholders define each prioritized outcome, their reasons to include or exclude them, and selected quotations.

**Table 3:** Mental health recovery outcomes prioritized by stakeholders, their definitions, and reasons to include or exclude them

Outcome	Definitions made by all stakeholders	Reasons to prioritize it	Quotations
Psychosocial functioning	Patients’ capacity to resume daily activities and social interactions, including work and study; improve their personal care; be aware of their condition; learn to handle their symptoms; and adhere to their treatment. Participants deemed that this outcome would be more influenced by the mental health treatment, since it depended on patients’ capabilities.	Policymakers: Since CMHC focuses on patients’ independence and right to work and study, recovery would be visible through their psychosocial functioning	“Psychosocial functioning allows us to measure the autonomy of the person, that he or she has a life project and that the person has the necessary tools so they can have a productive role within our society.” (Female policymaker)
		Workers: It is important to restore the areas that were affected due to the mental health condition	“When I see psychosocial functioning, I see that there is improvement, I feel that they are happy. That plays an important role in seeing if the person is recovering” (Male CMHC worker)
		Patients: Shows their autonomy, positive contributions to others, and capacities to face challenges	“I was able to start and finish a degree, I can express my emotions again, I’ve forgiven my family, my relationship with my parents is better, I love them very much” (Male patient with schizophrenia, Northern Lima)
Quality of life	General well-being and ability to carry out their daily activities, have healthy relationships, access to health and education services, good housing conditions, social reintegration, individual autonomy, and the adoption of healthy habits. Participants mentioned that this outcome was related to the objective living conditions of patients and would be harder to change due to mental health treatment alone, requiring a comprehensive approach with other institutions (e.g. labor sector, education, housing, food security, etc.)	Policymakers: Even though CMHC could promote the recovery of patients, this would not be sustainable if patients have a poor quality of life	“If we work on the quality of life of patients, we will come to perceive the best quality of life for them and their families. This will have a favorable impact on the years of life that would no longer be lost, both due to death and disability, and would also allow our country to be healthy” (Male policymaker)
		Workers: Relates to other areas of life, such as basic needs, satisfaction with material conditions, and how others perceive them (i.e. acceptance, stigma)	“Quality of life is based on the interaction of different areas of your life or of the person, it can be in the social, educational, or work environment and it will allow the person to have that feeling of well-being” (Male CMHC worker)
		Patients: It involves improving their current conditions, being accepted and connected to their communities, and reaching their dreams and personal aspirations	“I feel happy because I’m getting back to how I was before. Now I can work, I can attend meetings, I can be with my friends, I can be with my whole family. I’m recovering, taking my medications, I’m getting back on track” (Male patient with substance abuse, Ucayali)
Symptoms	Refers to the presence and evolution of physical and psychiatric symptoms associated with their mental health condition, and how patients learn and workers guide on how to maintain a healthy living despite them. They seek not only to their reduction but also to the acceptance of living with them without interfering with their daily lives.	Policymakers: The easiest outcome to assess and the first thing that changes when patients recover.	“The symptoms make it very difficult for the person to recover. (For example) they might hear voices, but if you don’t teach them to work through these voices, it could become more complex” (Female policymaker)
		Workers: Symptoms cause daily life difficulties and their reduction is the first step towards recovery.	“To at least begin to improve, there must be a decrease in their symptoms. If there is no decrease in their symptoms, they will hardly function well, they will have the same difficulties.” (Male CMHC worker)
		Patients: It is important to reduce them adhering to their treatments, but they were not too emphatic on its relevance.	“If I don’t follow my treatment, there’s an imbalance: I can’t sleep, I’m anxious, I hear voices. Yet, that only happens when I don’t take my medicines for a month. Now (…) I can talk about it, I’m able to differentiate reality from that other world” (Female patient with schizophrenia, Ucayali)
Emotional balance	Ability to maintain emotional and mental stability, freeing oneself from tension and finding peace in different aspects of their lives. It also meant trusting and loving themselves.	Patients: Sense of feeling good and satisfied with who they are, trusting their own capacities	“Before, my self-esteem was very, very, very low. I didn’t feel like doing much; I just wanted to lie on my bed and sleep all the time. With treatment, I’ve learned to love myself, respect myself, and take care of myself so that I can help others” (Female patient with depression, Southern Lima)
Personal growth	Have greater confidence in their own abilities, recognizing their own worth, and prioritizing their emotional well-being. It also involved the ability to learn from adversity and move on, as well as adopting an optimistic attitude towards life.	Patients: Process of improving and growing as an individual, facing and transforming challenges into opportunities. Mostly mentioned by patients with depression, anxiety and psychosocial issues	“(My recovery) began when I learned to forgive, because (…) I let go many things, I healed many things and that also made me improve as a professional, grow as a person, and now I feel that I have yet work to do, but it is no longer like at the beginning when I was at zero, now I am taking it step by step and I am feeling better (Female victim of violence, Arequipa)”

*CMHC* means community mental health center

#### Prioritized scales to measure the selected mental health recovery outcomes

The prioritization of recovery scales followed a sequential process. Additional file 5 presents detailed information about the prioritized scales. Table 4 shows how the initial set of scales identified in the systematic review and recommended by the mental health experts was reduced gradually, until the final selection of two scales: WHODAS-12 and DIALOG.

**Table 4:** Prioritization of scales by each stakeholder throughout the co-prioritization process

Outcome	Preselected scales by the research team	Scales prioritized by policymakers	Changes made by the MHDs heads	Scales prioritized by CMHC workers	Scales assessed by CMHC patients
Psychosocial functioning	CORE-OM				
	OQ-45				
	HoNOS	HoNOS			
	CANSAS-P	CANSAS-P	CANSAS-P		
			WHODAS-12* ^**+**^	**WHODAS-12** ^**+**^	**WHODAS-12** ^**+**^
Quality of life	SF-36	SF-36	SF-8*		
	EQ-5D				
	**DIALOG** ^**+**^	**DIALOG** ^**+**^	**DIALOG** ^**+**^	**DIALOG** ^**+**^	**DIALOG** ^**+**^
Symptoms	K10	K10			
	PHQ-9	PHQ-9	PHQ-9		
	GAD-7	GAD-7	GAD-7		
	SSQ-14			^b^	
	CGI				
			HoNOS*		
Shared decision-making	SDM-Q-9	SDM-Q-9			
	OPTION	OPTION			
	SDM-PROCESS-4		^a^		

*CMHC* Communitymental health centers, *MHD* Mental Health Direction, *CORE-OM* The Clinical Outcomes in Routine Evaluation-Outcome Measure, *OQ-45* Outcome Questionnaire-45, *HoNOS* Health of the Nation Outcome Scales, *CANSAS-P* The Camberwell Assessment of Need Short Appraisal Schedule, *SF-36* Short Form-36 Health Survey, *EQ-5D* European Quality of Life 5 Dimensions, *DIALOG scale*, *K-10* Kessler Psychological Distress Scale, *PHQ-9* Patient Health Questionnaire-9, *GAD-7* Generalised Anxiety Disorder Assessment, *SSQ-14* Shona Symptoms Questionnaire 14, *CGI* Clinical Global Impression, *SDM-Q* Shared Decision Making Questionnaire, OPTION scale, *SDM-PROCESS-4* The shared decision-making scale

* MHDs’ heads suggested to change, add and move these scales for the revision of CMHC workers and patients

^a^MHDs’ heads decided to take out these scales because they said that the shared decision-making outcome would be better suited as part of the quality assessment of mental health services, in charge of another health direction of the MoH

^b^CMHC workers did not prioritize any scale to assess symptoms

^+^ DIALOG and WHODAS, the two prioritized scales, are highlighted to show their transit throughout the co-prioritization process

Here, we describe the prioritization process followed with each stakeholder. First, based on the systematic review identified in component 2 (4 outcomes, 12 scales, see Table 2), and incorporating a series of scales suggested by the mental health experts (3 extra scales), the research team presented to policymakers a pre-selected list of scales corresponding to the outcomes that they prioritized in the first workshop (4 outcomes, 15 scales). Policymakers reviewed each of these scales in small groups and selected nine, to then be reviewed by CMHC workers and patients.

These scales were prioritized mainly based on their comprehensiveness to assess the outcome, length (brief to be able to use them in CMHC), ease of use, existing validations in the Peruvian context, and their adjustment to the community-based model. Reasons to exclude scales were that they did not measure the outcome they should (i.e. they mentioned that CORE-OM and OQ-45 measured more symptoms than psychosocial functioning), they were not aligned to the community-based model of care, or they were too long to introduce them in the CMHC routines.

Then, in the meeting with MHD’s heads, they made some changes to the prioritization of policymakers. They moved HoNOS from psychosocial functioning to symptoms, because they deemed that this scale focused more on symptoms than in the functioning of patients; changed the SF-36 to the shorter SF-8 to ease their usability in CMCH routines; added the WHODAS-12 to psychosocial functioning to maintain at least two scales per outcome since it is a brief and well-known scale that could be used in public health services; and took out the shared decision-making outcome because they preferred to include it into a different policy that the MHD was working on regarding the assessment of health services’ quality.

This new set of three outcomes and seven scales was then presented in the workshop with CMHC workers, who reviewed them and prioritized two scales: WHODAS-12 (Psychosocial functioning) and DIALOG (Quality of life). They said that DIALOG could be enough to measure both mental health outcomes, but still deemed important to consider WHODAS-12. Regarding psychosocial functioning, many workers considered CANSAS more comprehensive; however, WHODAS-12 was finally prioritized because they believed it was quicker to administer, it could be used for various diagnoses, and CANSAS’s terms would need to be clarified before using it. Regarding quality of life, DIALOG was selected unanimously by all workers because they said it was easy to understand, rapid to administer, comprehensive, and appropriate to assess quality of life. Yet, some workers stated that they should be cautious when using it due to potential biases in patients’ perceptions of their own wellbeing.

Finally, for assessing symptoms, workers did not select any scale because those presented were diagnosis-specific, and they preferred a short multi-diagnostic one. Instead, they recommended creating a new scale based on the checklist of symptoms that they currently use in the CMHC. In the meeting with MHD’s heads, they did not deem this appropriate because it was a checklist and did not show changes across time nor severity, and adding these would need a psychometric validation process. Thereby, no scale for symptoms was presented in the workshops with patients.

Lastly, during the four patients’ workshops, participants practiced completing the two prioritized scales and said that they felt comfortable responding to both, but a little better with DIALOG. They liked that DIALOG had a broader scope to assess several topics, and that WHODAS-12 was more specific to identify mental health issues without directing too much of the conversation. Patients agreed that WHODAS-12 and DIALOG could be both used in CMHC routines. Yet, they identified some difficulties in understanding some items of both scales, especially those patients with lower educational level, and those with schizophrenia, depression, and anxiety. For WHODAS-12 specifically, patients mentioned that items were too long, and the constant use of the word “difficulties” was confusing. For DIALOG, they had issues understanding the meaning of some specific wording (e.g. “practical help”, “personal safety”).

Participants provided a series of recommendations on the conditions to use these scales in the CMHC routines. For instance, policymakers said that most of these scales could be used at the beginning of patients’ treatment, as a baseline, and then every three to six months to assess changes. Some CMHC workers mentioned the same periodicity, whereas others said that scales should be aligned to the care packages, at the beginning, middle, and end of each package to assess changes. Policymakers mentioned the CMHC consulting rooms as the place to apply these scales, while workers added home visits and the waiting room as potential spaces.

Regarding the personnel in charge of using these scales, policymakers and most workers said that any trained worker could administer them, but some workers said that nurses would be the best option to use them at baseline since they are the first person in contact with patients. Patients did not state any preference, saying that they would feel comfortable responding to these scales by any CMHC worker.

## Discussion

This study aimed to co-prioritize a set of recovery outcomes and scales to assess the recovery of people living with mental health issues who receive care in CMHC of Peru. We conducted a three-component process, including constant communication with the MHD of Peru; a literature search and conversations with international experts; as well as workshops with key stakeholders: policymakers, CMHC workers, and patients. As a result, five recovery outcomes were co-prioritized: psychosocial functioning, quality of life, symptoms, emotional balance, and personal growth. Likewise, two scales were co-prioritized by all stakeholders: WHODAS-12, to assess psychosocial functioning, and DIALOG, to assess quality of life. The measurement of the three other outcomes remains as a pending task since key stakeholders did not agree on a single scale to assess the diversity of symptoms that CMHC patients present, and the last two outcomes emerged in the final phase of this process, during the workshops with patients, so that the other stakeholders did not discuss potential scales to assess them.

Similar to previous research in LMICs, we found that our participants conceive mental health patients’ recovery as a non-linear process that transcends the mental health services they use [33]. Participants understood recovery as a process in which they can experience both improvement and relapse in an iterative way, and even if they feel “recovered”, they would benefit from receiving further support to maintain it. Moreover, and aligned to experiences from other LMICs [33], we found that interpersonal relationships are an important element in their recovery, both as family support and social connectedness, since they not only mentioned the importance of receiving help but also sharing and enjoying life with them.

Regarding our specific outcomes identified, we found that even though psychosocial functioning and quality of life had similar contents, participants differentiated them based on the extent in which treatment could have an impact. Psychosocial functioning was more related to patients’ abilities, at an individual level, easier to be improved by their health treatments; whereas quality of life was more related to their social and material conditions, harder to modify by health treatment itself. This distinction aligns with current understandings of recovery, in which recovery involves both a personal change of the individual but also poses importance on the social environment and access to basic services [34]. For instance, an study conducted in Ecuador identified interpersonal relations, family support, and access to healthcare as relevant factors for recovery [35].

The outcome of symptoms, highly valued by policymakers and workers, was not rated as so important by patients; probably because patients focus more on how their daily lives are affected by these symptoms and not the symptoms per se. Other studies found that health workers are mainly focused on symptoms’ reduction [36, 37]; whereas other authors mentioned that patients do not assess their mental health based on their medicines intake or service use [38], possibly explaining why symptoms’ remission are deemed different by each stakeholder. The Mental Health Directorate reflected that workers and policymakers are trained to focus more on the medical diagnoses than the lived experiences of patients, so that their emphasis on considering symptoms as a necessary outcome.

Emotional balance and personal growth were emergent outcomes that patients prioritized and were not considered by policymakers nor workers. Similar to previous research, we found that patients interpreted their recovery as having their own agency, being self-sufficient, and gaining new knowledge about themselves and their mental health conditions due to their treatments [39]. These outcomes also aligned to previous recovery frameworks, such as the CHIME framework, which proposes five recovery processes in people with mental health issues: connectedness to peers, others, and the community; hope and optimism about the future, including their own recovery and positive thinking; identity, which involves redefining their identity into a positive lens and overcoming stigma; meaning in life, related to giving meaning to their experiences and setting goals; and empowerment, more focused on their strengths and having control over their lives [40].

Finally, shared decision-making was prioritized only by policymakers because they saw it as a result of the autonomy that a patient could gain due to their recovery. This is aligned with the community-based model of care that promotes the autonomy of mental health patients [8, 9]. However, this outcome was taken out because the MHD mentioned that it could be better assessed as part of other kinds of outcomes, showing that the MoH has other initiatives related to the assessment of mental health services [10].

Our results mirror the findings of a previous overview of systematic reviews and qualitative meta-synthesis on mental health recovery [34]. Authors did not place much importance on the reduction of symptoms, but on the acceptance of having a mental health issue and the skills on how to handle it (*symptoms outcome*). They also emphasized the need to address the social determinants of health, which we found are independent of the CMHC treatments (*quality of life*), and a sense of control, autonomy, and responsibility to manage their mental health condition (*psychosocial functioning*). Finally, recovery was a process of overcoming despair and gaining a sense of wellbeing (*emotional balance*) and a sense of purpose and meaning (*personal growth*).

Prioritized scales were selected mainly based on their extension, ease of use, comprehensiveness, and relation with the community-based care model. Some scales recommended by international sources (e.g. systematic review and experts’ opinions) were discarded by our participants due to these criteria, even though they were validated and had good psychological properties, such as PHQ-9, GAD-7, EQ-5D, among others. This evidences the interplay between scientific evidence and local conditions when designing a public health policy [19].

### Implications

The reflections made by our mental health experts, in terms of how workers would be incentivized or penalized, as well as patients encouraged to decide between recovering and leaving the health services, must be taken into account. Our participants offered guidance on how to contextualize the introduction of these recovery scales in CMHC by recommending who would administer them, where and how frequently. Indeed, the existing collaboration with the MHD may ease this implementation in real-world settings; however, as suggested by policymakers and health professionals from similar settings [41], it will be essential to allocate specific budget, intersectoral collaboration, and community commitment to assure it.

Moreover, the current implementation of electronic health records could be a good opportunity to introduce these scales as a standardized measure in CMHC nationwide since workers could have easier access to the scales and their results, better data storage and security, as well as less time spent per assessment [42]. Yet, the MoH must guarantee proper technological infrastructure, involve key stakeholders since early stages to assure their buy-in, provide specific training and support to CMHC workers, and engage patients to provide valid responses and be informed about their results [18].

### Next steps

Moving from the number of people treated to the assessment of relevant and meaningful outcomes for patients would be an important change in the Peruvian mental health system monitoring. Thereby, next steps for the MHD would be the selection of scales to assess the remaining outcomes to enable a comprehensive assessment of recovery in their mental health patients. For the assessment of psychiatric symptoms, this might include a co-production process with key stakeholders to design a scale broad enough to cover the great array of diagnoses treated in CMHC, though short enough to be able to incorporate them in their quotidian care. On the other hand, for the outcomes of emotional balance and personal growth, a literature search will be recommended as a first step to identify if existing scales could be incorporated in CMHC, and if possible, followed by a similar co-prioritization process.

The prioritized scales have good psychometric properties and have been validated in similar settings. WHODAS-12 has been recommended for populations with mental disorders and from different backgrounds [43]; whereas DIALOG was specifically designed for people with severe mental disorders and have shown acceptable psychometric properties [44] and been used in similar settings, such as Colombia [45]. Still, we recommend testing the implementation of these scales before scaling it up in Peru. This testing should include specific training to use the scales, a close clinical and technological support, and anticipate potential challenges, such as an increased burden to workers and patients, changes in the CMHC workflow, or concerns about data security and privacy [18].

### Strengths and limitations

The co-prioritization process described here can be replicated in other countries, especially LMICs, to define and adopt a set of mental health recovery outcomes and scales to be used in community mental health services. The iterative nature of this co-prioritization process allowed us to constantly reflect on the emerging results and to adjust the methodology when needed. Moreover, based on the fact that having the participation of different stakeholders, especially end-users, such as workers and patients, is a good practice and recommended when designing public health policies, we gathered the experiences of a wide range of stakeholders from different cities of Peru and who have vast experience with recovery in mental health patients. Our results can inform public policies in Peru, but generalization to other contexts might be limited. Other countries could also include this diversity of participants to assure a deeper comprehension of recovery and better tailor their results to their particular settings.

Despite having limited resources to reach large audiences, we reached a national scope by engaging with participants from different regions of the country (e.g. coast, highlands, and the Amazon) and people from different professional and lived-experience backgrounds. This was possible thanks to the collaboration with the Peruvian MHD, which promoted the inclusion of different voices and ease the access to these populations. However, having the voice of patients at the end of this process reduced our possibilities to discuss their emergent outcomes with the other stakeholders and to review appropriate scales for their assessment. Additionally, a potential selection bias could be present since we involved patients willing to participate and they may not represent the broader population. Yet, to ameliorate this potential bias, we sought to guarantee the participation of patients with different diagnoses and stages on their recovery processes.

Even though the research team collected information about potential outcomes and scales beforehand, the inductive design followed in the workshops with CMHC workers and patients allowed to collect their own experiences and then pair them with the existing evidence, instead of biasing and limiting their responses. Similar processes have been followed by researchers in other LMICs [46]. Worth noting, not all scales identified in the systematic review were included in the workshops because the research team could not access them (i.e. Beginning Services Survey-BSS), they were too long to be used in community settings (i.e. Symptom Checklist-90-R), they were used in hospitals and not community-based settings (i.e. Hospital Anxiety and Depression Scale), or because according to the review, they were rarely used (i.e. Social Functioning Scale). Nonetheless, participants identified some scales that matched their needs in CMHC and plan to incorporate them in their care practices.

## Conclusions

This study aimed to co-prioritize a set of recovery outcomes and scales to be used in CMHC routines involving policymakers, CMHC health workers and patients from different cities of Peru. As a result, five recovery outcomes were prioritized: psychosocial functioning, quality of life, symptoms, emotional balance, and personal growth. To assess these outcomes during CMHC routinely care, all stakeholders agreed on the selection of two scales: WHODAS-12, to assess psychosocial functioning, and DIALOG, to assess quality of life. The measurement of the three other outcomes is still a pending task. Next steps include incorporating and testing the use of the co-prioritized scales in CMHC routines, and introducing them into the electronic health records that the MoH is currently implementing in CMHC.

## Supplementary Information

Supplementary Material 1.

Supplementary Material 2.

Supplementary Material 3.

Supplementary Material 4.

Supplementary Material 5.

## Data Availability

The datasets generated during the current study are available from the corresponding author on reasonable request.

## Abbreviations

CMHC: Community mental health centers
LMIC: Low- and middle-income countries
MHD: Mental Health Directorate
MoH: Ministry of Health
