# Supplementary material for: Co-prioritization of mental health recovery outcomes and scales for community mental health centers in Peru
Source: BMC Health Serv Res. 2025 Sep 1;25:1162. doi: 10.1186/s12913-025-13140-7 (PMC12400696; doi:10.1186/s12913-025-13140-7)
Supplement: Supplementary file 1 — Supplementary Material 1. [file 12913_2025_13140_MOESM1_ESM.docx]

**Additional file 1: Timeline of the activities conducted in each component and their aims**

| **Month, 2023** | **Activity** | **Aim** |
| --- | --- | --- |
| January 30th | First meeting with heads from the MHD | Decide to conduct the co-prioritization of mental health recovery outcomes and scales |
| February - March | Literature search + Conversations with experts | Collect information about recovery outcomes and scales used worldwide |
| April 13th | First workshop with policymakers | Review the information collected about recovery outcomes used worldwide and prioritize those that could be used in CMHC in Peru |
| April 21st | Second meeting with heads from the MHD | Discuss findings of the first workshop and define the objectives and methods for the next ones |
| April 27th | Second workshop with policymakers | Review the information collected about recovery scales used worldwide and prioritize those that could be used in CMHC in Peru |
| May 10th | Third meeting with heads from the MHD | Discuss findings of the two workshops with policymakers and define the outcomes and scales that would be presented to CMHC workers |
| June 20th | First workshop with CMHC workers | Collect their experiences with recovery in mental health patients and select up to four mental health recovery outcomes |
| June 21th | Second workshop with CMHC workers | Review all scales selected by policymakers and prioritize up to two scales per outcome |
| July 11th | Fourth meeting with heads from the MHD | Discuss findings of the two workshops with CMHC workers and define the methods for the workshops with patients. Define the scales that would be presented to patients |
| July 20th | First workshop with CMHC patients (Southern Lima, Coast) | Identify their perceived recovery outcomes and review the scales prioritized by policymakers and workers |
| August 10th | Second workshop with CMHC patients (Northern Lima, Coast) |  |
| September 4th | Third workshop with CMHC patients (Arequipa, Highlands) |  |
| October 16th | Fourth workshop with CMHC patients (Ucayali, Amazon) |  |
| November 10th | Fifth meeting with heads from the MHD | Discuss findings of the workshops with policymakers, CMHC workers and patients and define the next steps for their implementation in CMHC nationwide |
