# Supplementary material for: Co-prioritization of mental health recovery outcomes and scales for community mental health centers in Peru
Source: BMC Health Serv Res. 2025 Sep 1;25:1162. doi: 10.1186/s12913-025-13140-7 (PMC12400696; doi:10.1186/s12913-025-13140-7)
Supplement: Supplementary file 2 — Supplementary Material 2. [file 12913_2025_13140_MOESM2_ESM.docx]

**Additional file 2**: Interview guideline with international mental health experts

| **INTERVIEW GUIDELINE WITH INTERNATIONAL MENTAL HEALTH EXPERTS** | | | |
| --- | --- | --- | --- |
| **Participant data** | | | |
| 1 | Sex |  | |
| 2 | Age |  | |
| 3 | Profession |  | |
| 4 | Nationality |  | |
| 5 | Area of mental health expertise (i.e. mental health reforms, monitoring and evaluation of mental health services) |  | |
| 6 | Experience with LMIC | 1 | Have worked with a LMIC |
|  |  | 2 | Have worked in a LMIC |
| **Interview guide** | | | |
| **Mental health outcomes** | | | |
| 1. What outcomes would you recommend to assess recovery in CMHC patients? 2. What are the gold standards to assess recovery in mental health patients? 3. What outcomes are used in the community-based mental health services of your country? 4. What outcomes are you informed that LMIC use in their mental health services? | | | |
| **Scales to asses mental health recovery outcomes** | | | |
| 1. What scales would you recommend to assess recovery in CMHC patients? 2. What scales are used in the community-based mental health services in your country? 3. What scales are you informed that LMIC use in their mental health services? 4. What recommendations would you have on how to use these scales in the CMHC of Peru? | | | |
