# Supplementary material for: Co-prioritization of mental health recovery outcomes and scales for community mental health centers in Peru
Source: BMC Health Serv Res. 2025 Sep 1;25:1162. doi: 10.1186/s12913-025-13140-7 (PMC12400696; doi:10.1186/s12913-025-13140-7)
Supplement: Supplementary file 3 — Supplementary Material 3. [file 12913_2025_13140_MOESM3_ESM.docx]

**Additional file 3: Topics covered in each workshop**

| **Participants** | **Workshop aims** | **Main contents** |
| --- | --- | --- |
| Policymakers | - Prioritization of recovery outcomes and scales - Discussion on experts’ opinions and literature review | - Overview of key recovery outcomes based on literature review and experts’ opinions. - Prioritization of recovery outcomes by small groups ranking them in order of relevance and significance. - Group discussion about the rationale behind the prioritization of selected outcomes. - Revision of recovery scales corresponding to the prioritized recovery outcomes. - Selection of scales by small group ranking them based on validity, usability, and applicability within the CMHC setting. - Group discussion about the rationale behind the prioritization of selected scales. |
| CMHC workers | - Reflection on experts’ opinion and literature - Prioritization of recovery outcomes and scales - Recommendations on the most suitable conditions to use the prioritized scales | - Collect CMHC workers' experiences identifying and assessing recovery in their patients. - Overview of recovery outcomes prioritized with policymakers, matching them with their own experiences. - Prioritization of recovery outcomes by group voting. - Revision of recovery scales corresponding to the prioritized recovery indicators. - Prioritization of recovery scales by group voting to rank them based on validity, usability, and applicability within the CMHC setting. - Group discussion about the rationale behind the prioritization of selected scales. |
| CMHC patients | - Collect their own experiences with recovery - Identification and prioritization of recovery outcomes - Review of prioritized recovery scales | - Collect CMHC patients’ experiences with their mental health condition, service use and recovery. - Explore their own recovery processes by drawing their journeys, highlighting improvements, challenges and desired future recovery goals. - Prioritization of recovery outcomes by selecting five key recovery outcomes. - Explore patients’ personal definitions of recovery and their perspectives on its attainability. - Revision of prioritized scales based on their easiness to understand, need for further explanation, and comfort with completing these scales. |
