# Supplementary material for: Co-prioritization of mental health recovery outcomes and scales for community mental health centers in Peru
Source: BMC Health Serv Res. 2025 Sep 1;25:1162. doi: 10.1186/s12913-025-13140-7 (PMC12400696; doi:10.1186/s12913-025-13140-7)
Supplement: Supplementary file 4 — Supplementary Material 4. [file 12913_2025_13140_MOESM4_ESM.docx]

**Additional file 4: Mental health outcomes recommended by experts, grouped based on the outcomes identified in the systematic review.**

| **Mental health outcomes identified in the systematic review** | **Specific mental health outcomes or categories recommended by experts** |
| --- | --- |
| 1. Psychosocial functioning | - **Community cohesion**: Connection with their community - **Working/Studying** or not (experts mentioned that this was meaningful to mental health patients) - **Participation** in community organizations or not - **Relationships**: Family and partner - **Living independently** |
| 2. Quality of life | - **Quality of life**: Many experts recommended this outcome, especially for people with severe mental disorders, because it could be used independently of the diagnosis, and because it is useful for economic studies. However, they also mentioned that results on this outcome would not show the changes made in symptoms, scales usually assess social conditions, which are harder to change as a result of a mental health treatment, and they did not identify a good-enough scale. |
| 3. Needs assessment | - **Relationships**: Family and partner - **Treatment adherence** |
| 4. Symptoms and symptoms severity | - **Symptoms**: All experts recommended this outcome and provided examples of potential scales, especially short ones and globally used, such as PHQ-9 or GAD-7. Some experts also recommended assessing this outcome by multi-diagnostic scales, such as BPRS; and others were cautious with this outcome since not all patients fall easily into specific diagnostics. - **Prevent relapse** |
| 5. Satisfaction with services | - Satisfaction with health services - Opportune care - Receiving care |
| 6. Disability and functional impairment | - Stigma: Current perception and changes over time |
