# Supplementary material for: Co-prioritization of mental health recovery outcomes and scales for community mental health centers in Peru
Source: BMC Health Serv Res. 2025 Sep 1;25:1162. doi: 10.1186/s12913-025-13140-7 (PMC12400696; doi:10.1186/s12913-025-13140-7)
Supplement: Supplementary file 5 — Supplementary Material 5. [file 12913_2025_13140_MOESM5_ESM.docx]

**Additional file 5: Information about prioritized scales to measure recovery in mental health patients**

| **Outcome** | **Preselected scales by the research team and/or MHD heads** | **Objective** | **Population** | **Scale** | **items** | **Scores** | **Administration** |
| --- | --- | --- | --- | --- | --- | --- | --- |
| **Psychosocial functioning** | Clinical Outcomes in Routine Evaluation-Outcome Measure (CORE-OM) | Self-reported questionnaire consisting of 34 items designed to assess the patient's condition across a series of dimensions: subjective well-being, problems/symptoms, functioning, and risk | Clinical | Four dimensions | 34 items | Mean scores for each dimension are calculated by dividing the total score by the number of completed responses corresponding to the items of each dimension. | Administered by the clinical professional |
|  | Outcome Questionnaire-45 (OQ-45) | The OQ-45 assesses three main dimensions designed to measure the patient’s overall condition in a therapeutic context: subjective distress, interpersonal relationships, and social role | Clinical and non-clinical. | Likert scale (5 points: Never to Almost Always) | 45 Items | Total score and three subscales: Subjective Distress (SD), Interpersonal Relations (IR), and Social Role (SR). | Self-report, administered by the clinical professional. |
|  | Health of Nation Outcome Scales (HoNOS) | The scale was designed to measure various physical, personal, and social problems associated with mental disorders. It is useful for measuring progress, change, or evolution, sensitive to improvement, deterioration, or the absence of change over time. | Clinical | Likert scale of 0-4. | 12 items | 0-48: Higher scores indicate greater severity. Scores are divided into four sections and show different patterns of evolution. The total score represents the cumulative severity of the different items, and can be used to measure evolution | Administered by the clinical professional |
|  | Camberwell Assessment of Need Short Appraisal Schedule (CANSAS-P) | Provide a comprehensive assessment of patients with severe mental health issues, including the evaluation of their needs | Clinical | Four multiple-choice response options: No need, Need met, Need unmet, Don't know / No response | 22 | Each item represents a need, and the rating of each item is independent. | Self-report, interviewer-administered (in-person or by phone), or through a proxy. |
|  | WHO Disability Assessment Schedule (WHODAS-12) | A generic and practical assessment scale that can measure health and disability in the population and in clinical practice. WHODAS 2.0 captures the level of functioning across six life domains: understanding and communication, mobility, self-care, relationships, daily activities, and participation | Clinical and non-clinical. | - Dichotomous scale (yes/no)  - Multiple choice: "mild," "moderate," "severe," and "extreme." | 36 items (12 in the brief version) | Scores assigned to each question [“none” (1), “mild” (2), “moderate” (3), “severe” (4), and “extreme” (5)] are summed. | Self-report, interviewer-administered (in-person or by phone), or through a proxy |
| **Quality of life** | SF-36 Health Questionnaire (Abbreviated SF-8 Version) | The SF-36 captures all relevant aspects to characterize an individual's health, through eight dimensions: physical function, physical role, bodily pain, general health, vitality, social function, emotional role, and mental health | Clinical and non-clinical | Multiple choice | 36 items (9 items in the abbreviated Spanish version) | For each of these dimensions, scoring scales can be computed, which are easily interpretable, all of which are ordered such that the higher the score, the better the health status. | Self-report |
|  | EuroQol 5-Dimension (EQ-5D) | To assess health-related quality of life based on five dimensions: mobility, self-care, usual activities, pain/discomfort, and anxiety/depression. | Clinical | Five dimensions with three response levels for each (no problems, some problems, and extreme problems). | 5 Items | Score from each dimension, with a summary score (EQ Index) based on utility values for each health state. | Self-report or proxies. |
|  | DIALOG | Used to measure satisfaction with life, aspects of treatment over time, and associated factors. Addresses eight domains of life (mental health, physical health, employment situation, housing, leisure, partner/family, friendship, personal safety) and three aspects of treatment (medication, practical help, meetings with healthcare professionals). | Clinical in community settings | Likert scale of 1-7, ranging from “completely dissatisfied” to “completely satisfied,” with a neutral point at 4. | 11 items | Each item represents a category of analysis and receives a unique rating from 1 to 7 | Self-report, administered by the clinical professional. |
| **Symptoms** | Kessler Psychological Distress Scale (K10) | To assess the level of psychological distress in a population based on anxiety and depression symptoms. | Clinical | Likert scale (5 points: none of the time to all of the time). | 10 items | Total score ranging from 10 to 50, with higher scores indicating higher distress levels. | Self-report |
|  | Patient Health Questionnaire (PHQ-9) | Used to detect the presence and severity of depression. | Clinical | Likert scale of 0-3 | 9 items | The total sum of responses suggests different levels of depression. Scores range from 0 to 27. In general, a total score of 10 or more suggests the presence of depression. | Self-report |
|  | Generalized Anxiety Disorder 7 (GAD-7) | Self-administered questionnaire to detect and measure the severity of generalized anxiety disorder | Clinical | Likert scale of 0-3 | 7 items | Evaluates the risk of experiencing nonspecific psychological distress (symptoms of anxiety or depression) in the past month. The evaluation is indicated by the total score, which is obtained by summing the scores from the seven items | Self-report |
|  | Social Support Questionnaire (SSQ-14) | To assess the emotional and instrumental support available to an individual. | Clinical and non-clinical | Likert scale (4 points: none of the time to all of the time). | 14 items | Total score for emotional and instrumental support, with a higher score indicating greater support. | Self-report, administered by the clinical professional. |
|  | Clinical Global Impression (CGI) | To evaluate the overall severity of the patient's condition and improvement over time, based on severity and improvement scores | Clinical | 7-point Likert scale for severity, and 7-point scale for improvement. | 1 items | Severity score from 1 (normal) to 7 (most severe); Improvement score from 1 (very much improved) to 7 (very much worse). | Administered by the clinical professional. |
| **Shared decision- making** | Shared Decision-Making Questionnaire (SDM-Q-9) | To assess the degree of shared decision-making in healthcare settings, specifically focusing on nine items related to the decision-making process. | Clinical | Likert scale (5 points: strongly agree to strongly disagree). | 9 items | Total score ranging from 9 to 45, with higher scores indicating more shared decision-making. | Self-report, administered by the clinical professional. |
|  | Observing Patient Involvement in Decision Making (OPTION) | To measure the level of shared decision-making between patients and clinicians in healthcare settings, focusing on patient involvement. | Clinical | Likert scale (0–4) rating specific aspects of decision-making. | 12 items | Total score ranging from 0 to 48, with higher scores indicating greater shared decision-making. | Administered by the clinical professional. |
|  | Shared Decision-Making Process (SDM-PROCESS-4) | To assess the process of shared decision-making in healthcare settings, focusing on the steps involved in making decisions. | Clinical | Likert scale (1–5) based on four steps in the decision-making process. | 4 items | Total score ranging from 4 to 20, with higher scores indicating better shared decision-making. | Administered by the clinical professional. |
